# Scanning methodology for contact lens-type ocular in vivo dosimeter (CLOD) dosimetry applying a silicone material

**DOI:** 10.1186/s13014-022-02056-9

**Published:** 2022-05-07

**Authors:** Jaeman Son, Jin Dong Cho, Seongmoon Jung, Chang Heon Choi, Jong Min Park, Jung-in Kim

**Affiliations:** 1 Department of Radiation Oncology, Seoul National University Hospital, Seoul, 03080 Republic of Korea; 2 Institute of Radiation Medicine, Seoul National University Medical Research Center, Seoul, 03080 Republic of Korea; 3 Biomedical Research Institute, Seoul National University Hospital, Seoul, Republic of Korea; 4 Department of Radiation Oncology, College of Medicine, Yonsei University, Seoul, Republic of Korea; 5 Robotics Research Laboratory for Extreme Environments, Advanced Institute of Convergence Technology, Suwon, Republic of Korea; 6 Department of Radiation Oncology, Seoul National University College of Medicine, Seoul, Republic of Korea

**Keywords:** Contact lens-type ocular in vivo dosimeter, Newton’s ring artifact, Scanning methodology

## Abstract

**Purpose:**

Contact lens-type ocular in vivo dosimeters (CLODs) were recently developed as the first in vivo dosimeter that can be worn directly on the eye to measure the dose delivered to the lens during radiotherapy. However, it has an inherent uncertainty because of its curved shape. Newton’s ring effect inevitably occurs because the spacing between the glass window and the active layer is not constant. Furthermore, it involves a large uncertainty because the objective of the CLOD with such morphological characteristics is to measure the dose delivered to an out-of-field lens. In this study, we aimed to investigate the effects of various compensating materials on the sensitivity, accuracy, and uniformity of analysis using a curved CLOD. We developed a new scanning methodology that involves applying a compensating material to reduce the uncertainty caused by the air gap.

**Methods:**

Four compensating materials—Dragon Skin™ 10 (DS), a transparent silicon material, SORTA-Clear™ 40 (SC), optical grease (OG), and air (no compensating material)—were used in this study. The CLOD was scanned in the reflective mode and transmission mode using each compensating material. We then examined the sensitivity, accuracy, and scan uniformity to evaluate the scanning methodology using compensating materials.

**Results:**

The increase in sensitivity was the highest for OG compared to that for air in the reflective mode. On average, the sensitivity in the reflective mode was higher than that in the transmission mode by a factor of 2.5 for each dose. Among the four compensating materials, OG had the smallest uncertainty. Therefore, the best scan uniformity was achieved when OG was used.

**Conclusions:**

Scanning methodology was proposed in which a compensating material is applied for a curved lens-type dosimeter. Our results show that OG is the most suitable compensating material to obtain the best accuracy of dose analysis. Following this methodology, the scan uncertainty of curved dosimeters significantly decreased.

## Introduction

**Fig. 1:**
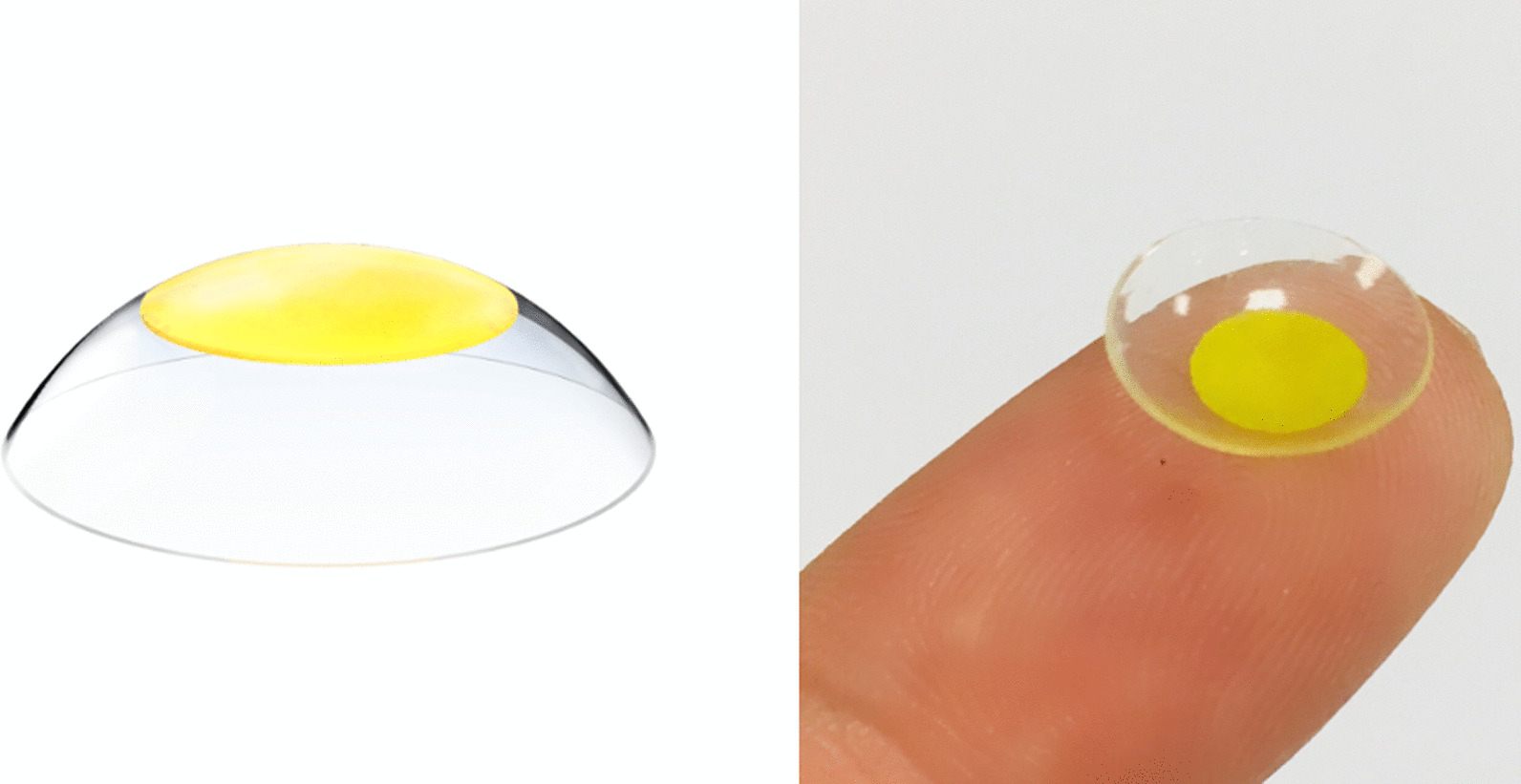
*CLOD* image

Contact lens-type ocular in vivo dosimeters (CLODs) have recently been developed as the first in vivo dosimeters that can be directly worn on the eye to measure the dose delivered to the lens during radiotherapy. Its physical properties are independent of energy and dose rate; furthermore, it does not depend on angular and scanning orientations, which can be a significant advantage for utilization as an in vivo dosimeter [1]. Another advantage of CLOD is that it has been verified for biological stability through cytotoxicity, sensitization, and eye-irritation tests. As shown in Fig. 1, a curved CLOD can be directly worn on the eye because it has a sandwich structure with an active layer fabricated of the lithium salt of pentacosa-10, 12-diyonic acid (LiPCDA) wrapped around silicone. LiPCDA is used to manufacture Gafchromic films. Color of LiPCDA in Gafchromic films changes when irradiated, and the delivered dose can be determined using this method. The transmission or reflection mode of a flatbed scanner is generally used for the quantitative analysis of CLOD, and in vivo dosimetry analysis using CLOD is performed in the same way as Gafchromic film analysis [2–5].

**Fig. 2:**
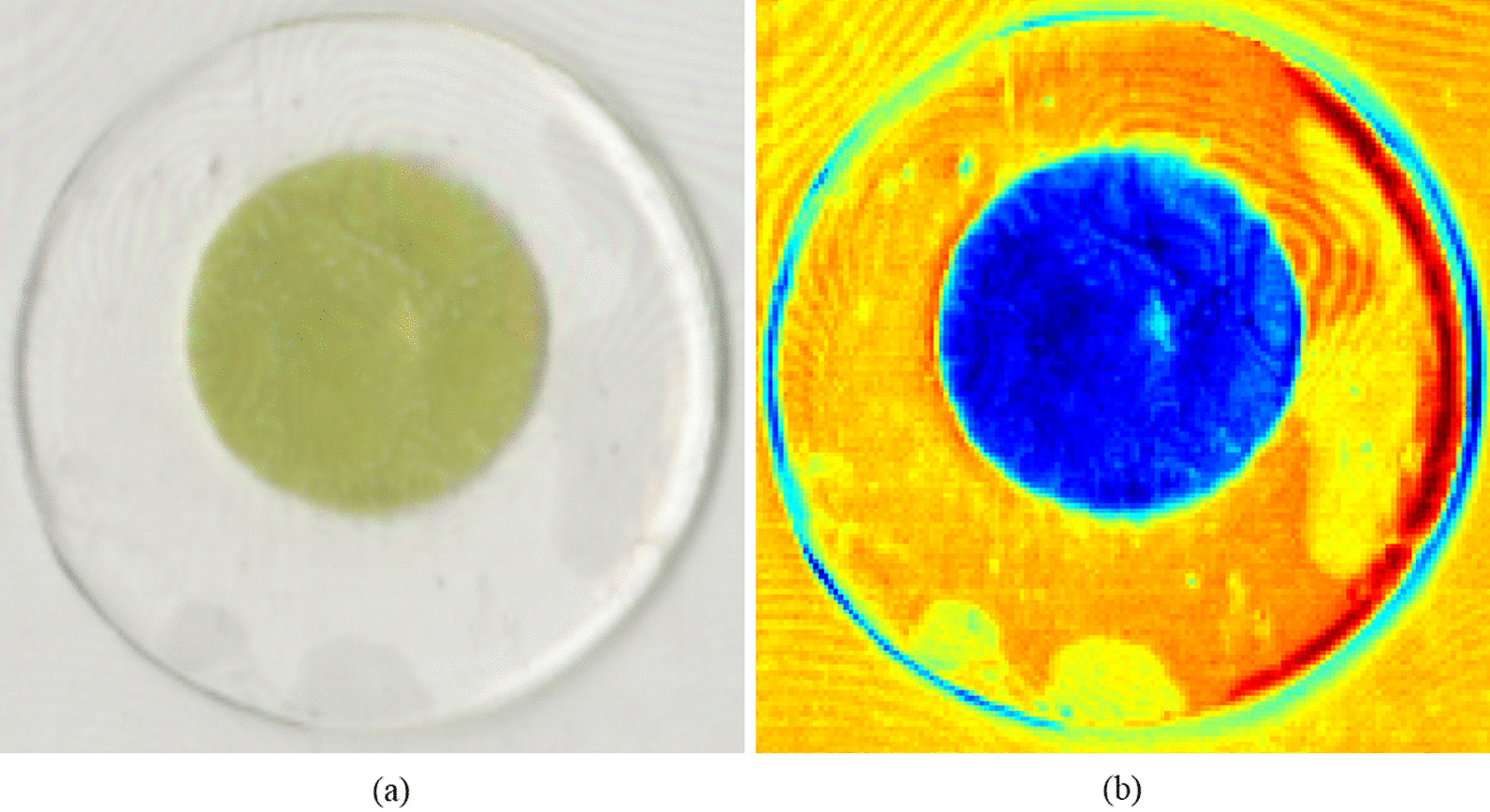
Newton’s ring artifact of CLOD: **a** scan image and **b** red channel image using a flatbed scanner

Papaconstadopoulos et al. reported that the flatbed scanner analysis of a dosimeter such as a film can be significantly affected by the scan protocol [6]. The red channel in the reflection mode at the lowest dose level achieved a sensitivity of up to 150% higher than that of the red channel in the transmission mode. Furthermore, they reported that the sensitivity, uncertainty, and accuracy of the scanning resolution and color channels of scanned images can also change. Because the material used in CLOD resembles a film, the same characteristics were considered in the analysis. Furthermore, additional items are considered for CLOD analysis because of their curved shape, unlike flat films. Generally, the contact between the transparent glass on the scanner and the object has a significant effect on film analysis, which is used to evaluate the dose based on the amount of light. Kairn et al. reported that Newton’s rings can be generated if the contact between the film surface and scanner surface is not even [7]. They reported that in a high-dose region, Newton’s rings cause an uncertainty of ± 5% (± 1 SD) and up to 30% uncertainty in the low-dose area of out-of-field regions. CLOD also generates a Newton’s ring artifact when analyzed with a flatbed scanner, as shown in Fig. 2.

However, CLOD has inherent uncertainty because of its curved shape. The Newton’s ring effect inevitably occurs because the spacing between the glass window and the active layer is not constant. Furthermore, because the objective of the CLOD, with such morphological characteristics, is to measure the dose delivered to an out-of-field lens, it inevitably has a large uncertainty. Therefore, we aimed to develop a new scanning methodology to analyze the effect of applying a compensating material to increase the sensitivity and reduce the uncertainty caused by the air gap for a curved CLOD.

## Materials and methods

To investigate the new scanning methodology, four compensating materials were used: Dragons Skin™ 10 (DS, Smooth-On, Macungie, PA, USA), a transparent silicon material, SORTA-Clear™ 40 (SC, Smooth-On, Macungie, PA, USA), BC-630 optical grease (OG, Saint-Gobain Korea, Korea), and air (no compensating material). The CLOD was scanned in the reflective mode and transmission mode using each compensating material spread on a glass of Epson 10000XL (Epson Seiko Epson Corp., Nagano, Japan) flatbed scanner. A flatbed scanner, is the most commonly used device for performing film dosimetry, has a dependency for position. So, we used the mask based on acrylic material in order to ensure consistency for position of CLOD in flatbed scanner. After the scan of CLOD, we used alcohol swabs to clean and then analyze the surface whenever the compensating materials were changed. The RGB image collected from a 16-bit depth per color channel at a spatial resolution of 300 dpi was saved in the tiff format. After scanning, the pixel value of each CLOD was obtained, and the net optical density (netOD) was determined according to the following equation:

1 $$\mathrm{netOD}=\mathrm{OD}-{\mathrm{OD}}_{0}= {\mathrm{log}}_{10}(\frac{{M}_{exp}-{M}_{bkg}}{{M}_{unexp}-{M}_{bkg}})$$

where ${M}_{unexp}$ is the pixel value before beam irradiation, ${M}_{exp}$ is the pixel value after beam irradiation, and ${M}_{bkg}$ is the pixel value without an object.

The analysis software RIT113 v.7.71 (Radiological Imaging Technology, Inc., Colorado Springs, CO) was used for the pixel value, and the region of interest (ROI) was set to 3.5 × 3.5 mm^2^. This corresponds to about 40 × 40-pixel readout (300 dpi). To evaluate the scanning methodology using compensating materials, we examined the sensitivity, accuracy, and scan uniformity. The dose was irradiated using the Varian TrueBeam system (Varian Medical Systems, Palo Alto, CA), and doses in the range of 0–100 cGy were delivered with an energy of 6 MV in the reference condition based on the TG-51 protocol proposed by the American Association of Physicists in Medicine (AAPM). Each CLOD was irradiated three times for each dose.

To determine the sensitivity, we obtained a dose–response curve for each compensating material by evaluating the average pixel value using the irradiated CLOD for each dose. These curves were fitted to the power law function of Eq. 2 to obtain the parameters. This procedure was performed in each scan mode.

2 $$\mathrm{netOD}=a\bullet D+b\bullet {D}^{n}$$

where a, b, and n are the fitting parameters, and D is the measured dose (Gy). Then, the CLOD sensitivity was defined by the derivative of the slope of the dose–response curve at each point as follows:

3 $$\mathrm{S}= \frac{dnetOD}{dD}=a+n\bullet b\bullet {D}^{n-1}$$

where a, b, and n have the same meaning as in Eq. 2.

The calibration curve was obtained using the measured OD for the dose irradiated to the CLOD. When the dose was measured using the CLOD, the measured unknown dose was a function of the measured netOD and was determined using the fitting curve. Hence, the measured netOD was converted to a dose value using a dose–response curve. This analysis calculation formula provides the result by fitting the data using the least-squares method. The following equation uses dose as a dependent variable:

4 $$D=a\bullet netOD+b\bullet net{OD}^{n}$$

Calibration was performed at a dose of 0–100 cGy using Eq. 4. The curve was obtained by power law fitting, where a, b, and n are the fitting parameters and D is an unknown dose in Gy. Devic et al. performed an uncertainty analysis to separate the uncertainty contribution of the fitted calibration curve and experimental contribution [8]. Equations 5–7 below represent the dose uncertainty analysis. The relative experimental uncertainty of the measured dose is given by Eq. 5 as follows:

5 $${\sigma }_{{D}_{exp}}\left(\%\right)=\frac{\sqrt{{(b+n\bullet c\bullet net{OD}^{n-1})}^{2}\bullet {\sigma }_{netOD}^{2}}}{{D}_{fit}} \times 100$$

where ${\sigma }_{netOD}^{2}$ is the uncertainty of film scanning. Furthermore, the relative fit uncertainty is expressed as follows:

6 $${\sigma }_{{D}_{fit}}\left(\%\right)= \frac{\sqrt{net{OD}^{2}\bullet {\sigma }_{b}^{2}+ {netOD}^{2\bullet n}\bullet {\sigma }_{c}^{2}}}{{D}_{fit}} \times 100$$

where ${\sigma }_{b}$ and ${\sigma }_{c}$ denote the fitting parameter uncertainties. The total relative uncertainty of the measured dose is expressed as follows:

7 $${\sigma }_{{D}_{tot}}\left(\%\right)= \frac{\sqrt{{netOD}^{2}\bullet {\sigma }_{b}^{2}+ {netOD}^{2\bullet n}\bullet {\sigma }_{c}^{2}+ {(b+n\bullet c\bullet {netOD}^{n-1})}^{2}\bullet {\sigma }_{netOD}^{2}}}{{D}_{fit}} \times 100$$

To evaluate the accuracy, doses of 25 and 70 cGy were applied to the CLOD. Then, the absolute doses for the transmission mode and reflective mode were evaluated using the calibration curve for each material, and the difference between them was determined.

Finally, to evaluate scan uniformity, the average coefficients of variation (CV) were calculated using the standard deviation (SD) and the average pixel value in the ROI for each irradiated CLOD of the dose–response curve, and the results were compared.

## Results

**Fig. 3:**
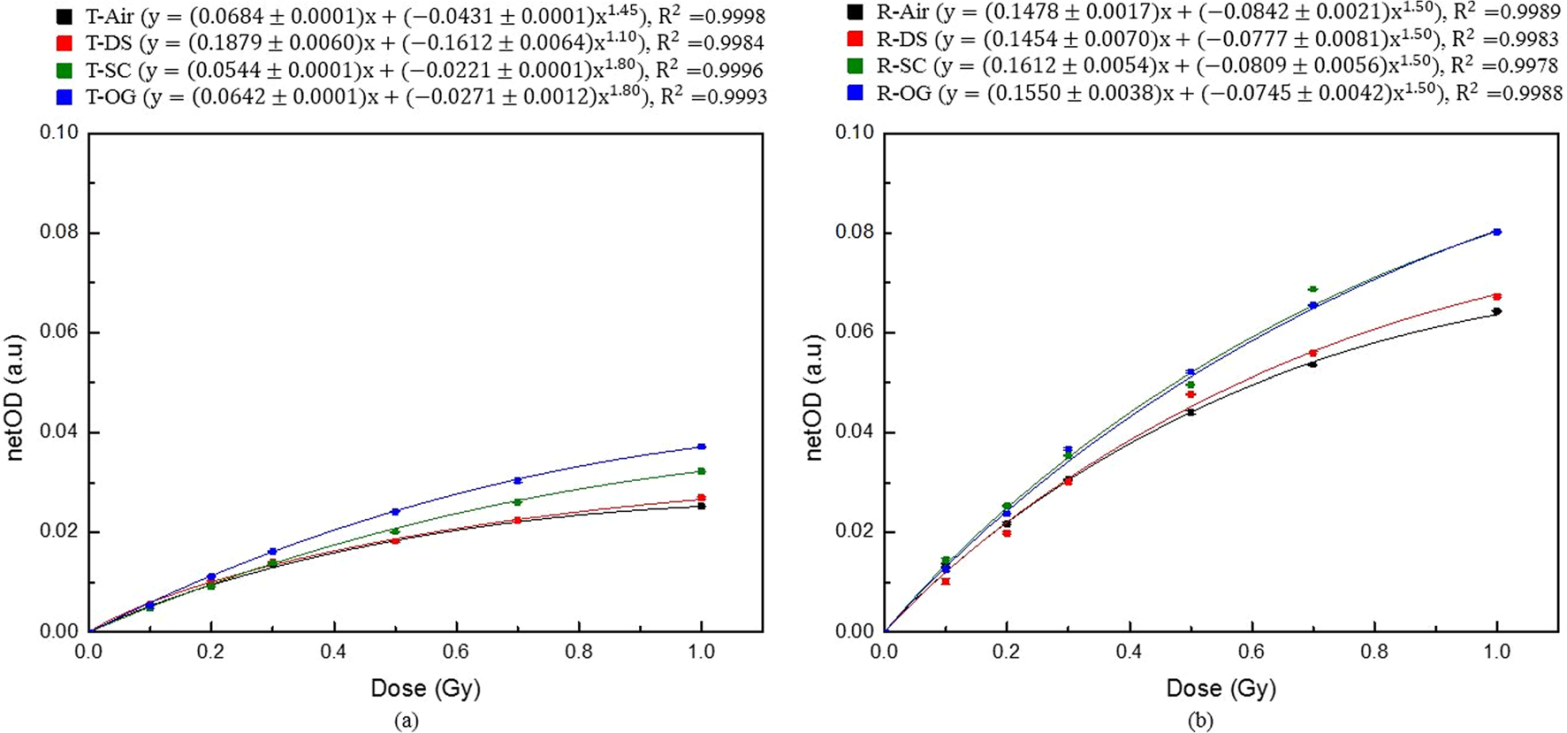
Dose–response curves for CLOD according to the compensating material in the dose range of 0–100 cGy: **a** transmission mode and **b** reflective mode

The measured netOD corresponding to the compensation material was determined from the pixel value at different dose values using the formula proposed by Devic et al. [8]. Figure 3 shows the dose–response curve of the CLOD for doses in the range of 0–100 cGy in each scan mode for each compensating material. Figure 3a shows the dose–response curve for the transmission mode, and Fig. 3b depicts the result for the reflective mode. Power law fitting was performed using Origin software (OriginPro 8.5.0 SR1, OriginLab Corporation, Northampton, MA, USA). Curves were fitted with a power of n, and the fitting parameters were determined according to the compensating materials for the dose–response curve in Fig. 3. The goodness of fit was determined based on adjusted R^2^ values. The R^2^ value tends to 1 as the fit approaches the data points. This fitting parameter was used to calculate the sensitivity.

**Fig. 4:**
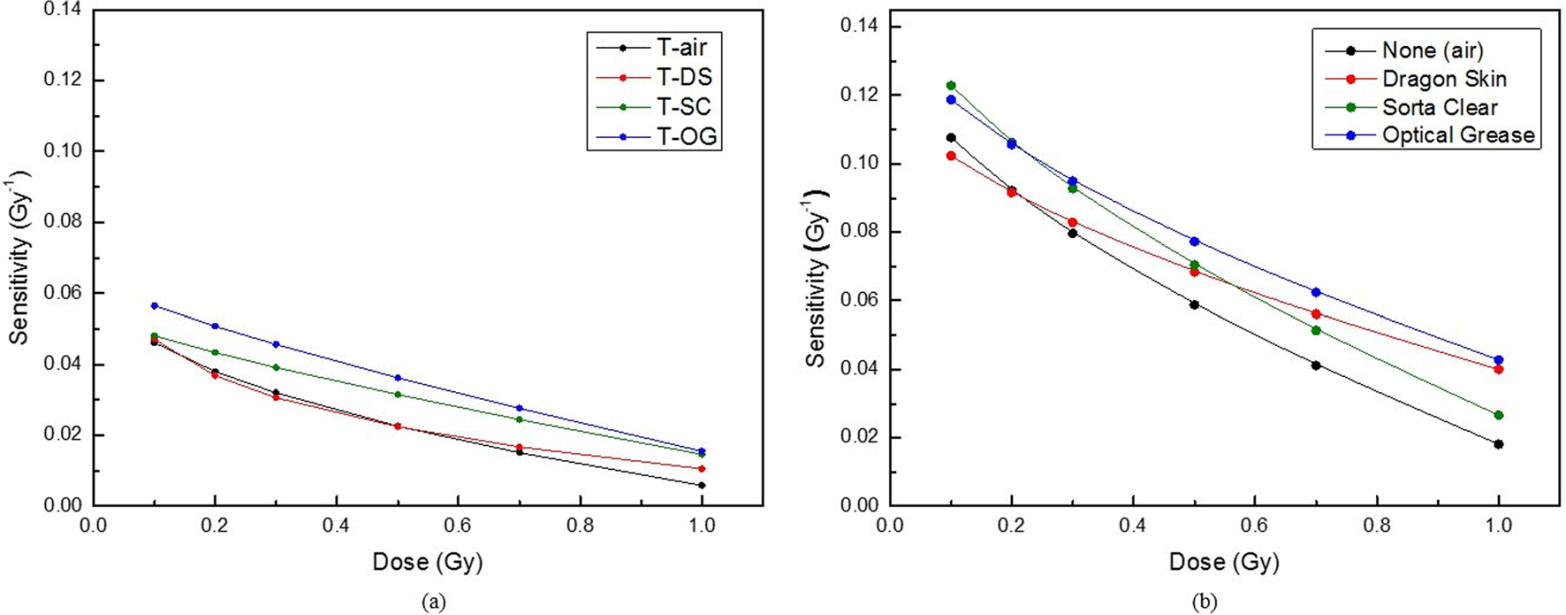
Sensitivity curves of CLOD according to the compensating material in the dose range of 0–100 cGy: **a** transmission mode and **b** reflective mode

Figure 4 shows the sensitivity curves for each compensating material in the dose range of 0–100 cGy. Figure 4a, b show the result of the transmission and reflective mode, respectively. The sensitivity was calculated using Eq. 3 for each delivered dose by inputting the values of a, b, and n determined using the dose–response curve. The sensitivity linearly decreased for each compensating material.

Figure 4 shows that the sensitivity at 50 cGy increased by -0.8%, 39.5%, and 60.4% for DS, SC, and OG, respectively in the transmission mode, compared with air. Furthermore, the increase in sensitivity was the highest for OG compared with air in the reflective mode. The sensitivity increased by 7.6%, 28.9%, and 29.9%, respectively, for DS, SC and OG. Furthermore, the sensitivity was the highest for other doses as well as for 50 cGy when OG was used as the compensating material regardless of the scan mode. On average, the sensitivity in the reflective mode was higher than that in the transmission mode by a factor of 2.5 for each dose. The compensating materials do not directly affect the sensitivity of the dosimeter. Since radiochromic films using flat scanners are analyzed using light transmittance, however, the compensating materials can cause light loss and thus change the sensitivity for compensating materials.

**Fig. 5:**
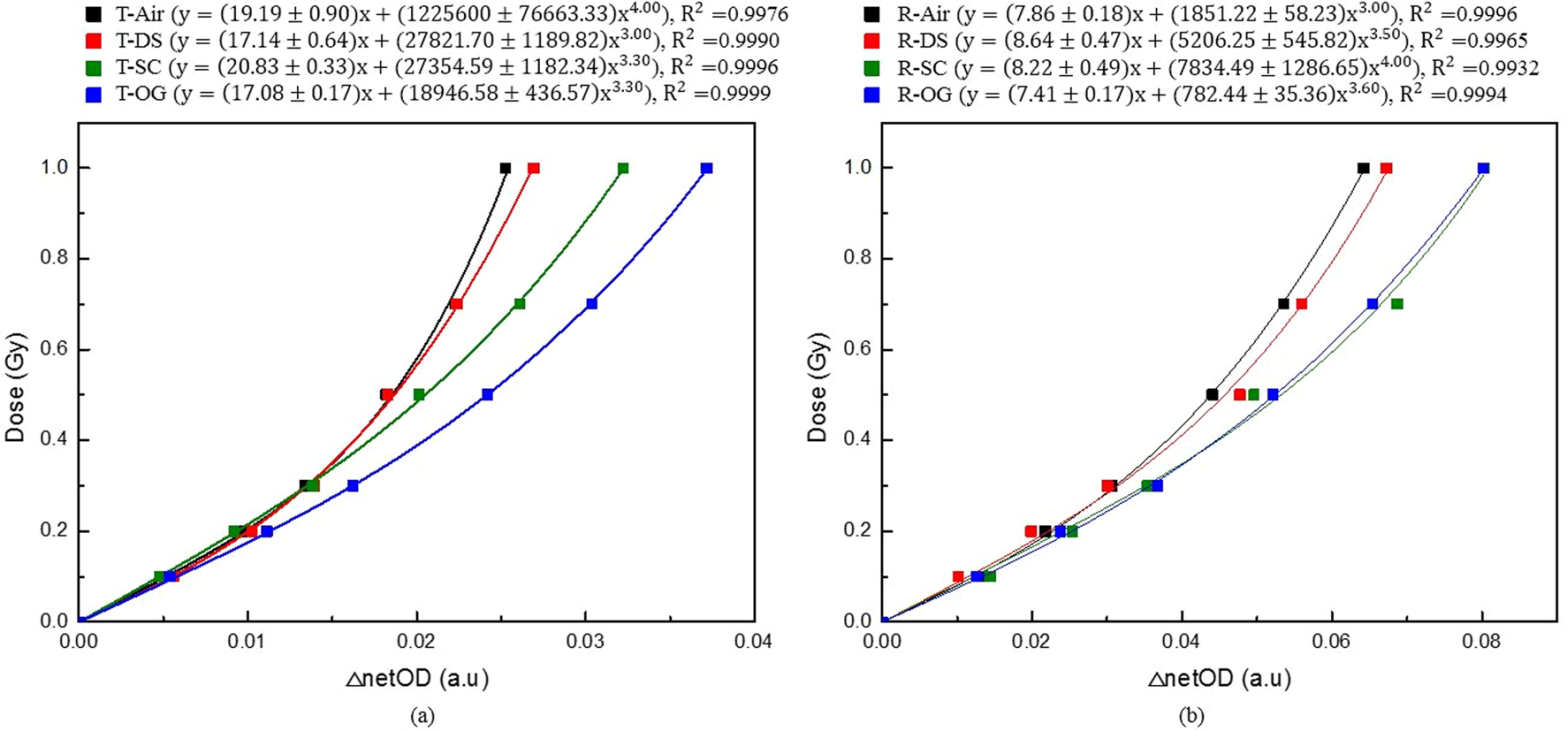
Calibration curves of CLOD according to the compensating material in the dose range of 0–100 cGy: **a** transmission mode and **b** reflective mode

**Fig. 6:**
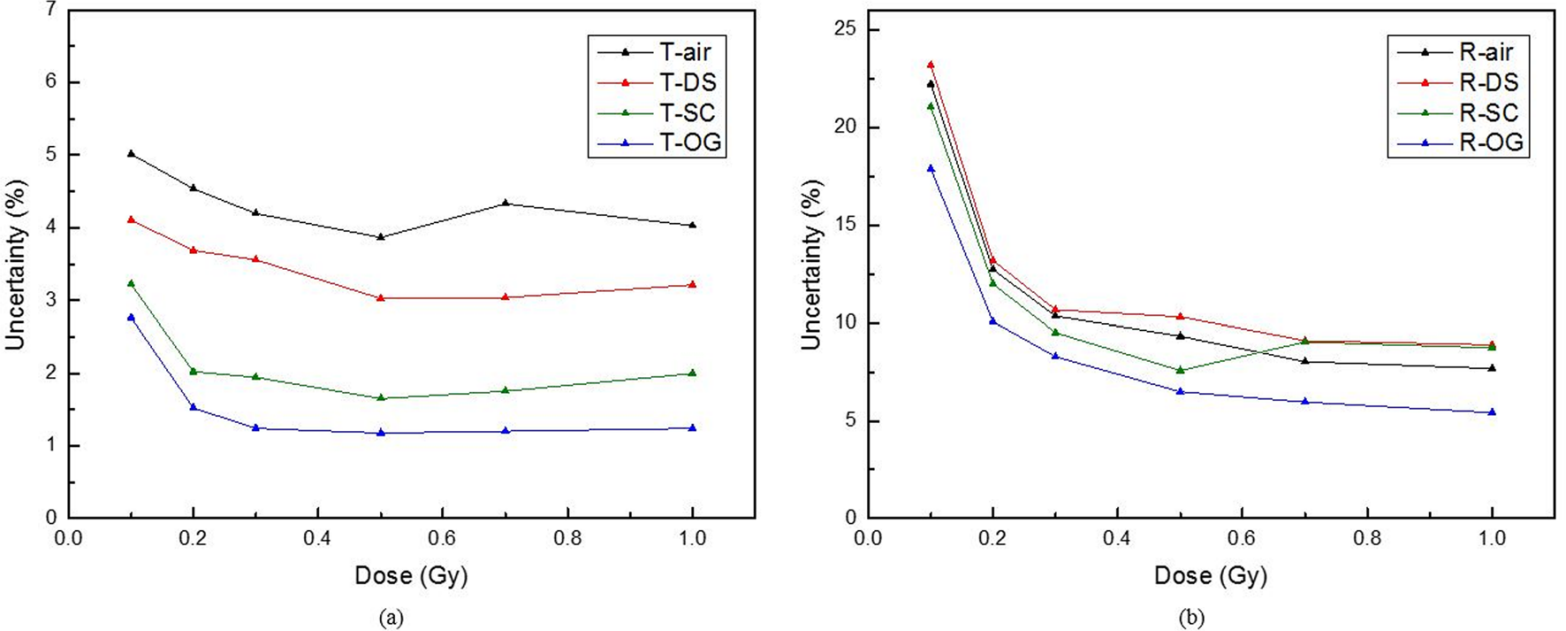
Uncertainty curves of CLOD according to the compensating material in the dose range of 0–100 cGy: **a** transmission mode and **b** reflective mode

Figure 5 shows the calibration curve for each compensating material in each scan mode obtained using a 6 MV photon beam for the CLOD. The calibration curve for each netOD was obtained based on Eq. 4. The unknown dose by the netOD measurement was estimated using this calibration curve. When the unknown dose was determined, the total uncertainty was calculated using Eq. (6). The calculation results are shown in Fig. 6. At 50 cGy, the uncertainties of air, DS, SC, and OG were 3.9%, 3.0%, 1.7%, and 1.2%, respectively, in the transmission mode, and 9.3%, 10.3%, 7.6%, and 6.5%, respectively, in the reflective mode. Thus, we observed that among the four compensating materials, OG had the smallest uncertainty.

In addition, the accuracy was examined using the calibration curve for each of the four compensating materials, and the results are listed in Table 1. The pixel value SD of air, DS, SC and OG for 25 cGy were 766.3, 696.5, 695.0 and 611.9 in transmission mode, respectively. In reflective mode, the pixel value SD were 577.1, 495.9, 492.9 and 373.3. These tended to be the same for 70 cGy. The SD of air, DS, SC and OG in transmission mode were 757.7, 727.7, 726.4 and 655.4. The pixel value SD were 589.9, 462.1, 458.0 and 405.5 in reflective mode. As a result, it was confirmed that the pixel value SD of OG was the lowest for both scan mode. At doses of 25 and 70 cGy, the dose difference for air in the two scan modes was the largest at 6.1% on average, while that for OG was the smallest at 1.0% on average. Furthermore, at doses of 1 Gy or lower, the dose difference of scanning in the transmission mode was larger than that in the reflective mode for all compensating materials. These results mean that the best scanning method is to analyze using OG in reflective mode to perform absolute dosimetry using CLOD.

**Table 1:** Accuracy results of CLOD according to the compensating material in the transmission and reflective modes

Delivered dose	25 cGy				70 cGy			
Scan mode	Transmission mode		Reflective mode		Transmission mode		Reflective mode	
Materials	Dose (cGy)	Dose diff. (%)	Dose (cGy)	Dose diff. (%)	Dose (cGy)	Dose diff. (%)	Dose (cGy)	Dose diff. (%)
Air	26.7	6.8	26.3	5.2	74.6	6.6	74.1	5.9
DS	26.4	5.4	23.8	4.8	74.5	6.4	73.9	5.5
SC	23.7	5.4	26.2	4.7	73.6	5.2	73.4	4.9
OG	25.6	2.2	25.3	1.3	70.2	0.3	70.1	0.2

Table 2 presents the results of scan uniformity. It shows the CV obtained using the average pixel and the SD values in the ROI depending on whether or not the material adhered to the glass surface in each scan mode and for each compensating material. The result is similar to the accuracy; the CV was smaller in the reflective mode than in the transmission mode. In the reflective mode, the values of air, DS, SC, and OG were 0.0134, 0.0110, 0.0115, and 0.0097, respectively. Thus, the results show that the best scan uniformity was achieved when OG was used.

**Table 2:** Results of scan uniformity of CLOD according to the compensating material in the transmission and reflective modes

	Air	DS	SC	OG
Transmission mode	0.0166	0.0161	0.0162	0.0146
Reflective mode	0.0134	0.0110	0.0115	0.0097

## Discussion

In this study, we investigated the effects of various compensating materials on the sensitivity, accuracy, and uniformity of analysis when using a curved CLOD in clinical practice. Based on the results, we found that the compensating materials help reduce the effect of Newton’s ring artifacts when scanning the CLOD. The CLOD indicates similar properties to an EBT film because they are both fabricated using LiPCDA. Numerous studies have reported on the various properties of EBT films, including those focusing on analysis methods and dose verification. Ferreira et al. reported a reading protocol for an EBT film that can be applied to IMRT verification by using an Epson flatbed scanner [9]. Subsequently, Gotanda et al. reported that using a flatbed scanner in the reflective mode enhanced the precision in the low-dose region below 100 mGy [10]. Papaconstadopoulos et al. suggested a protocol that uses the reflective mode with a red channel in the measurement of an EBT3 film dose of 2 Gy or lower [6]. Generally, the CLOD developed to measure lens doses also follows a similar method. Kim et al. analyzed CLOD in the reflective mode for the out-of-field low-dose region and reported that it can be used clinically. They noted that Newton’s ring artifacts could occur because of the curved shaped of the CLOD. A Newton’s ring artifact, which is also observed in EBT2, is a phenomenon that occurs when the contact between the film and the glass of the flatbed scanner is uneven. In EBT3, the Newton’s ring artifacts were minimized by adding microscopic silica to make the distance between the glass and film constant [11–14]. However, this approach is not available because CLOD is geometrically different from EBT3 film. To address this problem, Kim et al. spread the OG evenly on a flatbed scanner and then closely attached the CLOD to it. However, they did not explain how to use the optical grease. Therefore, we quantitatively determined the effects of the four silicone materials on CLOD analysis. Based on the findings of this study, we recommend a scanning methodology that applies OG when CLOD is used.

Emami et al. reported that if the lens delivered a dose of more than 10 Gy, the risk of cataracts is 5% within/after 5 yr (TD 5/5) for conventional prescription [15]. According to M. Kamrava et al., if a cumulative dose is delivered to the lens of 24 Gy or more, the 5-year cumulative incidence of radiation-induced cataracts was 92% compared with 65% in those receiving less than 12 Gy [16]. Radiation-induced cataracts are usually treated with standard surgical techniques to improve vision. If vision does not improve through surgery, comorbidities such as radiation retinopathy, vitreous hemorrhage, retinal detachment, or optic neuropathy should be suspected. Consequently, in order to reduce the incidence of radiation-induced cataracts or other comorbidities, the dose delivered to the lens must be sufficiently considered in treatment planning process. However, even if considered in the treatment planning process, it is difficult to accurately calculate the lens dose because the volume is very small and charged particle equilibrium is not satisfied in a superficial region of the body. To overcome the lack of electronic equilibrium, Monte Carlo simulation is essential to accurately calculate the deposited dose to the lens. As the Monte Carlo simulation is very time consuming in treatment planning process, however, it is difficult to use it clinically. It is also necessary to reflect the beam delivery errors and errors caused by the movement of the eyeball during the treatment. Therefore, there is a need for a method capable of accurately evaluating the dose clinically delivered to the lens. Kim et al. already reported that the results of in vivo dosimetry were compared with the TPS, MC calculations and CLOD measurements with a human phantom. They confirmed that the calculated value of Monte Carlo simulation was better matched with the measured value using CLOD than the calculated value of TPS. Therefore, it is necessary to accurately measure the dose delivered to the lens using CLOD. In addition, how the scanning methodology is performed has a significant impact on dose measurements. The scanning methodology suggested in this paper has the advantage of minimizing the effect on newton's ring artifact. Moreover, there is no need to purchase additional equipment for CLOD exclusive readout system because flatbed scanners are already installed in many hospitals.

All of our data indicated that a flatbed scanner can measure the effective OD of the CLOD. Real OD measurements of the CLOD are necessary to utilize the CLOD for absolute dosimetry. However, as the CLOD is only a relative dosimeter for lens dose measurements, it is sufficient to have a calibration curve obtained using effective OD measurements instead of the real OD measurements. Instead, when using CLOD clinically using effective OD, uncertainty in each dose should be considered. The clinically acceptable errors for prescription dose recommended by AAPM is 5%. This is only error for the dose delivered to the tumor and dose not correspond to clinically acceptable errors for normal organs. Because normal organs usually receive the low doses, it is not common to indicate a relative error at a low dose region. For example, 5% for 100 cGy is 5 cGy, whereas 5% for 10 cGy is 0.5 cGy. Therefore, as shown in Fig. 6, the uncertainty of the dose was indicated, and it is recommended to use this as a reference. After establishing scanning methodology for the analytical method, we will measure the doses by using the CLOD in a clinical study and compare these with TPS and Monte Carlo simulations.

## Conclusions

We proposed scanning methodology for analyzing the effects of applying a compensating material to increase the sensitivity and reduce the uncertainty for a lens-type dosimeter with curvature. Of compensating materials, OG is the most suitable compensating material in terms of the sensitivity, accuracy and uncertainty of dose analysis. The scan uncertainty of curved dosimeters can be significantly reduced following such scanning methodology.

## Data Availability

The datasets supporting the study conclusions are included within this manuscript.

## Abbreviations

CLOD: Contact lens-type ocular in vivo dosimeters
DS: Dragon Skin™
SC: SORTA-Clear™
OG: Optical grease
LiPCDA: Lithium salt of pentacosa-10, 12-diyonic acid
netOD: Net optical density
ROI: Region of interest
AAPM: American Association of Physicists in Medicine
CV: Average coefficients of variation
SD: The standard deviation
